# Effect of napabucasin and doxorubicin via the Jak2/Stat3 signaling pathway in suppressing the proliferation of neuroblastoma cells

**DOI:** 10.1590/acb396624

**Published:** 2024-09-30

**Authors:** İlker Ünlü, Mehmet Cudi Tuncer, İlhan Özdemir

**Affiliations:** 1 Beykent University – Faculty of Medicine – Department of Brain Surgery – Istanbul – Turkey.; 2 Dicle University – Faculty of Medicine – Department of Anatomy – Diyarbakir – Turkey.; 3 Atatürk University – Department of Gynecology and Obstetrics – Faculty of Medicine – Erzurum – Turkey.

**Keywords:** STAT3 Transcription Factor, Cell Culture Techniques, Cell Proliferation, Neoplasms, Brain

## Abstract

**Purpose:**

Napabucasin (NP) is a natural compound that can suppress cancer cell proliferation and cell cycle by inhibition of the signal transducer and activator of transcription 3 (STAT3) gene. We examined the effects of NP on the proliferation and invasion of neuroblastoma cells (SH-SY5Y).

**Methods:**

Human neuroblastoma SH-SY5Y cell line was used in this study. NP was administered to groups at the doses of 0.3–1 µM. Cell viability was analyzed by MTT assay. Real-time quantitative reverse transcription polymerase chain reaction and western blot analysis assessed the expressions of interleukin (IL)-6 dependent Jak2/Stat3 signaling pathway. The MTT cell viability method was applied to determine the antagonistic-synergistic effects and inhibitory concentration (IC_50_) doses of doxorubicin (DX) and NP.

**Results:**

It was determined that 0.3–1 µM doses of NP killed the cells almost completely after 48 hours, and also that Jak2/Stat3 expressions decreased dose-dependently via IL-6. At the protein level, NP and DX were found to reduce Jak2 and Stat3 levels.

**Conclusions:**

NP showed that it suppresses the proliferation of neuroblastoma cells. Due to its inhibitory effect on Jak2 and Stat3, it can be used to prevent invasion of SH-SY5Y cells. NP, which can inactivate Jak2/Stat3, can be used as a treatment agent by combining with DX in proliferation pathway in neuroblastoma.

## Introduction

Suppressing the proliferation of neuroblastoma cells is a critical focus in pediatric oncology due to the aggressive nature of this cancer, which originates from neural crest cells. Key mechanisms driving neuroblastoma proliferation include genetic mutations, such as MYCN amplification, and dysregulated signaling pathways, including the Ras/MAPK and PI3K/Akt pathways. Targeted therapies aimed at these pathways, along with epigenetic modifiers, have shown promise in reducing tumor growth. For example, MYCN inhibitors and PI3K/Akt pathway blockers can disrupt critical survival signals in cancer cells. Additionally, emerging treatments such as immunotherapy, involving monoclonal antibodies and CAR-T cell therapy, provide new avenues for targeting neuroblastoma-specific antigens.

Combining these strategies with conventional chemotherapy and radiotherapy offers a comprehensive approach to managing neuroblastoma, highlighting the importance of integrated treatment regimens in improving patient outcomes^1^^,^ 2. In the clinic, its treatment is tried with chemotherapy, radiotherapy, immunotherapy, and surgical methods^3^. Despite the trials of many anti-cancer drugs and all the methods used in treatment, neuroblastoma is a type of cancer whose proliferation and migration are difficult to prevent^4^^,^ 5. Since existing chemotherapeutic drugs cause cell death but are insufficient to stop invasion, innovative approaches are needed to prevent invasion. Innovative therapeutic approaches aimed at inhibiting the migration of cancer cells are expected to specifically reduce drug-related toxicity and improve patient survival. There are studies in the literature showing that organic phenolic compounds provide effective results in cancer studies and may constitute an important target for these innovative approaches^6^^,^ 7.

Natural compounds obtained from many different sources, especially plants and animals, are important resources in drug research and development for cancer. Their large number and structural differences make them stand out in pioneering discoveries and make them a gift of nature. Quinonoid monomers such as naphthoquinone, phenanthrenequinone, benzoquinone, and anthraquinone are widely found in higher and lower plant species, and their anticancer activities have been also demonstrated^8^. Anthracyclines (such as adriamycin and daunorubicin), which have been widely used in many cancer treatments for years, are derived from anthraquinone^9^.

Napabucasin (BBI608), a natural naphthoquinone, is isolated from *Ekmanianthe longiflora, Newbouldia laevis*, and *Handroanthus impetiginosus*. It is a currently molecule in clinical trials for cancer treatment due to its broad-spectrum anticancer activities. It is a small molecule, and its method of administration is oral^10^. Napabucasin is an original molecule discovered to phosphorylate and suppresses the signal transducer and activator of transcription 3 (Stat3) up to pStat3, which controls important mechanisms such as growth, proliferation, and survival of cancer cells. It has been also reported that it plays a role as a cancer stem inhibitor by regulating the microenvironment^11^. Recent studies have found that this molecule is a substrate of NAD(P)H:quinone oxidoreductase 1 (NQO1) and functions as a drug that can bioactivate NQO1^12^.

The aim of this study was to investigate the effects of napabucasin and doxorubicin on suppressing the proliferation of neuroblastoma cells through the Jak2/Stat3 signaling pathway. Specifically, we sought to elucidate the molecular mechanisms by which these agents inhibit cell growth and assess their potential synergistic effects in targeting this pathway. The effects of napabucasin and doxorubicin, which are commonly used in chemotherapy, on the proliferation and invasion of SH-SY5Y cells were investigated.

## Methods

### Cell culture

The human SH-SY5Y neuroblastoma cell line was used in the study. It was cultured in EMEM and F12 medium mixed in a 1:1 ratio containing 10% fetal bovine serum (FBS), 2 mM L-glutamine and 1% penicillin/streptomycin at 37°C temperature, 95% humidity and 5% CO2. The culture medium was changed every 24 hours until the cells reached the required majority. Cell counting was done with a Roche device. Ten μl of cells suspended in 1 mL of medium and 10 μL of 0.2% Trypan blue dye were mixed, and 10 μL of the mixture was applied to the device apparatus and counted. Napabucasin (MedChem Express, New Jersey, United States of America) was applied to the cells at concentrations of 0.3–1 µM.

### Cell viability

MTT test was performed for cell survival (viability) analysis after incubation. For this purpose, the yellow tetrazolium MTT (3-(4, 5-dimethylthiazolyl-2)-2,5-diphenyltetrazolium bromide) test solution, prepared at the dose of 5 mg/mL, was pipetted into all wells at 20 µL/well. Then, the plates were left to incubate for 4 hours. After the incubation, the medium in the wells was completely removed, and 200 µL ultra puree DMSO (Merk, United States of America) was added to each well and waited in the incubator under dark conditions for 2–4 hours. At the end of this period, the plates were read spectrophotometrically at 492 and 650 nm wavelengths with a Multiskan GO microplate reader (ThermoScientific, United States of America). The value obtained from the vehicle-treated control group was determined as a comparative viability rate based on 100% viability.

### Determination of antagonistic-synergistic effect

In the study, stock solutions of doxorubicin and napabucasin agents were prepared using 50:50 V:V ultrapure ethanol (Merck, United States of America) and ultrapure water. One mM stock solutions were made for both agents, portioned and stored at -20ºC. In the applications, the final concentration of the vehicle in the flasks or plate wells was reduced to 0.1%.

To determine the antagonistic-synergistic effects and inhibitory concentration (IC_50_) doses of doxorubicin and napabucasin, the SH-SY5Y cell line was cultivated in 96-well culture dishes (plate) with 80 µL with a multiple digital pipette at the density of 5 × 10^3^ cells/well. The cells were incubated overnight to ensure adhesion, and single and combined drug application was made the next morning. In order to determine the antagonistic-synergistic effect between the two substances, eight different doses of the drugs were applied in 64 different doses with serial dilution, starting from 10-µM concentration, with a dosing square. The plates were incubated for 48 hours. Each dose-response frame was performed in one 96-well plate, and the experiment was repeated five times. Using the data obtained as a result of MTT analysis, the antagonistic-synergistic effects of the agents were determined with Combenefit (Cambridge, United Kingdom) software.

### Cell morphology, nuclear structure, and apoptotic body formation

In the project, nuclear morphology changes and apoptotic structures occurring after apoptosis caused by doxorubicin and napabucasin agents were determined in the SH-SY5Y cell line with NucBlue Live ReadyProbes Reagent (ThermoScientific, United States of America) specific dye. In this context, SH-SY5Y cell lines were planted in 24-well plates at 5 × 10^4^ cells/well, and the cells were incubated at 37°C and 5% CO_2_ conditions. The next day, vehicles were applied to these wells, doxorubicin (DX) IC50, napabucasin (NP) IC50 and DX + NP1 (0.7 + 0.3 µM) and DX + NP2 (0.7 + 1 µM) agents were applied to these wells using the IC50 values obtained in the 48-hour application. At the end of the application, staining was performed directly as live cell staining in accordance with the kit protocol, and the cells were incubated at 37°C for 30 minutes. At the end of this period, the plates were photographed using the Thermo EVOS FL ImagingSystem using bright field mode and fluorescence mode and a DAPI filter at 20x objective magnification.

### qRT-PCR analysis

SHSY-5Y cells (1 × 10^5^ cells/2 mL medium) were seeded in a six-well cell plate. Cells were incubated for 24 hours at 37°C temperature, 95% humidity and 5% CO_2_ environment. Napabucasin (0.3 and 1 µM) at appropriate doses to the cells was applied to the wells and incubated again for six hours. During the isolation phase, Purelink RNA mini kit (Thermo, United States of America) was used, and the kit protocol was followed. RNA samples (high-capacity cDNA reverse transcription kit, Applied Biosystem) were converted into cDNA according to the kit procedure. cDNA amounts were determined with Take3 Plate (Epoch Spectrophotometer System, Biotek). Jak2, Stat3, and interleukin (IL)-6 expression analysis were performed. Quantitative polymerase chain reaction (PCR) analysis was performed with TaqMan probe mixed solution (Taqman Probe-based technology, Applied Biosystem). The Βeta actin gene was used as an endogenous control. Optical PCR plates were used for real-time quantitative reverse transcription PCR (qRT-PCR) analysis, and each group was studied in three replicates. Results were calculated with the 2-ΔΔCt method15 and are presented as fold change compared to the control group. In the study, the expression levels of IL6/Jak2/Stat3 pathway genes in control and application groups of SH-SY5Y human neuroblastoma cells were analyzed by qRT-PCR method. The primers used to investigate the changes in the expression of these genes are in Table 1, in 5’-3’ order.

**Table 1:** Primers of genes analyzed by quantitative reverse transcription polymerase chain reaction method in 5’-3’ order.

IL6: F: ATGAACTCCTTCTCCACAAG, R: AGAGCCCTCAGGCTGGACTG
IL6-R: F: CAGCTGAGAACGAGGTGTCC, R: GCAGCTTCCACGTCTTCTTGA
JAK 2: F: CAGTGGTCAAGAGGGAAACA, R: TGTCTGAGCGAACAGTTTCC
STAT3 : F: GGAGGAGTTGCAGCAAAAAG, R: TGTGTTTGTGCCCAGAATGT
β-Actin: F: CCTCTGAACCCTAAGGCCAAC, R: TGCCACAGGATTCCATACCC
GAPDH;F:CGGAGTCAACGGATTTGGTCGTAT,R:GCCTTCTCCATGGTGGTGAAGAC

Source: Elaborated by the authors.

### Western blot

For each of the control and treatment groups, cells were planted in T75 flasks in three replicates, and drug applications were made after the cells adhered to the bottom of the flask. At the end of the incubation period, cells were collected for protein isolation. For protein isolation, first, lysis buffer was prepared by adding 10 μL of phenyl methyl sulfonyl fluoride (PMSF) solution, 10 μL of sodium orthovanadate solution, and 10 μL of protease inhibitor to each 1 mL of RIPA lysis buffer. A thin bead (NextAdvance, United States of America) with the diameter of 0.2 mm was placed in the cell pellets, and lysis was performed in a tissue disintegrator for 1 minute at 50 rpm. Then, 350-μL RIPA lysis buffer was added to the tubes and lysis continued. After the tubes were incubated in the shaker block at 4°C and 350 rpm for 5 minutes, the cell lysates were centrifuged at 4°C and 14,000 rpm for 15 minutes. Supernatants were transferred to new 1.5-mL volume tubes, and protein amounts were measured in mg/mL with OptizenNano Q (Mecasys, Korea). The isolated proteins were stored at -80°C until use.

Protein levels in the study were JAK2 (6-D3) antibody (NBP2-66913), phospho-Jak2 [p Tyr1007, p Tyr1008] antibody (SY24-03), STAT3 (9D8) antibody (NBP2-22471), phospho-STAT3 [p Tyr705] antibody (NBP2-24463) (NovusBio, United States of America), and β-actin antibody (ACTN05 (C4)). They were determined by Western blotting using ThermoFisher Scientific primary antibodies. First, SDS-PAGE electrophoresis was performed on the samples, then they were transferred to a nitrocellulose membrane and blocked with blocking solution. Then, incubation with antigen-specific primary antibody and secondary antibody was performed, respectively, and imaging was performed with the chemiluminescence method (ChemiDoc MP System). The results were analyzed with the program of the imaging system.

## Results

### Cytotoxic effect

**Figure 1:**
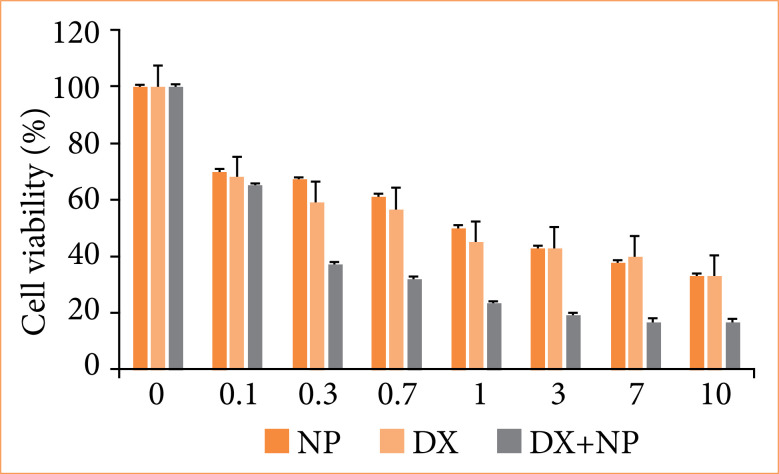
Effect of napabucasin (NP) and doxorubicin (DX) at different concentrations on the survival rate of neuroblastoma cells SH-SY5Y.

Source: Elaborated by the authors.

The effects of napabucasin on the proliferation of SH-SY5Y neuroblastoma cells were determined by the MTT assay cell viability detection method. It was determined that control group cells increased for 72 hours. It was observed that NP started to significantly reduce SH-SY5Y cell numbers from the moment it was first applied. As a result of DX and NP application, it was observed that there was a significant decrease in cell viability depending on the dose increase. Especially after the IC50 dose was determined (NP IC50 2.10, DX IC50 0.769), a very rapid decrease in cell viability was observed (Fig. 1).

### Determination of IC50

**Figure 2:**
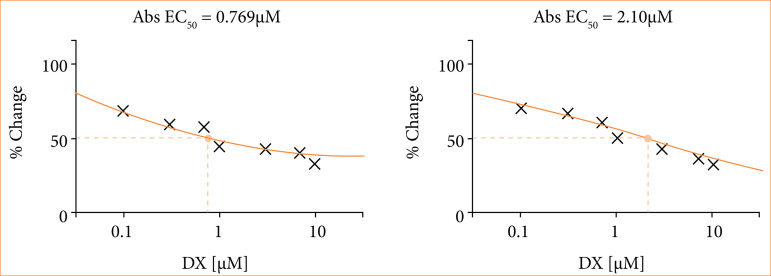
In SH-SY5Y neuroblastoma cell lines, the effect of doxorubucin and napabucasin (NP) agents on cell viability, which were applied in eight different doses using serial dilutions starting from 10 µM concentration for 48 hours, was determined by the results of the MTT test and the probit analysis using combenefit software.

Source: Elaborated by the authors.

In the first step of the study, to determine the IC50 doses of DX and NP applications on human neuroblastoma cell lines, SH-SY5Y cells were treated with eight different agent doses in 0–10 µM concentration ranges for 48 hours, and then the death rates were determined compared to the vehicle-treated control group using the MTT test. IC50 values determined for DX and NP during the 48-hour application period are given in Fig. 2.

### Determination of antagonistic-synergistic effect

**Figure 3:**
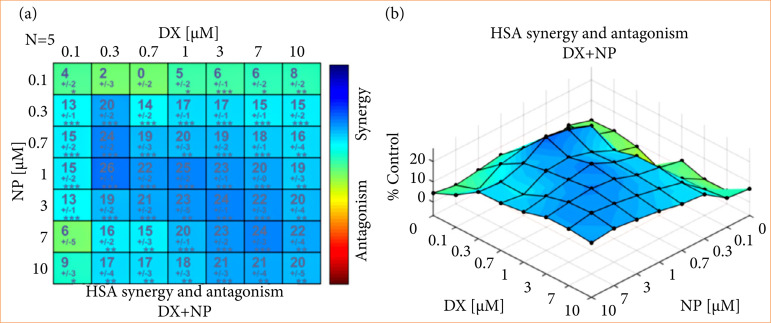
SH-SY5Y neuroblastoma cell lines. **(a)** Antagonistic and synergistic effect HSA ratios for doxorubicin (DX) + napabucasin (NP) of 64 different doses of DX and NP application with dosing square starting from 10-µM concentration for 48 hours. **(b)** Three-dimensional antagonistic synergistic score distribution.

Source: Elaborated by the authors.

In the study, DX and NP applications had a dose-dependent death effect on SH-SY5Y neuroblastoma cell lines. In combined applications, NP added to DX application strongly reduced the DX dose required for the death effect on neuroblastoma cancer cells. This effect was significantly evident in all combined doses of DX + NP, especially when both agents were administered alone. It was observed that doses below the IC50 doses per head showed a synergistic effect with HSA scores of 20.24 and 26% at DX + NP 0.3 + 0.3–0.7 and 1 µM doses, respectively, when applied in combination (Fig. 3). This effect was also found to be statistically significant.

Likewise, at a dose of 0.7 µM, corresponding to the IC50 dose of DX, the addition of 0.3 and 1-µM NP caused an additional mortality effect of 14 and 12%, respectively. These results showed that combined applications of the two agents could produce a high synergistic effect on SH-SY5Y neuroblastoma cell lines (Fig. 3). In the light of these data, the combined application doses were determined as DX + NP1 (0.7 + 0.3 µM) and DX + NP2 (0.7 + 1 µM) in the following parts of the study.

**Figure 4:**
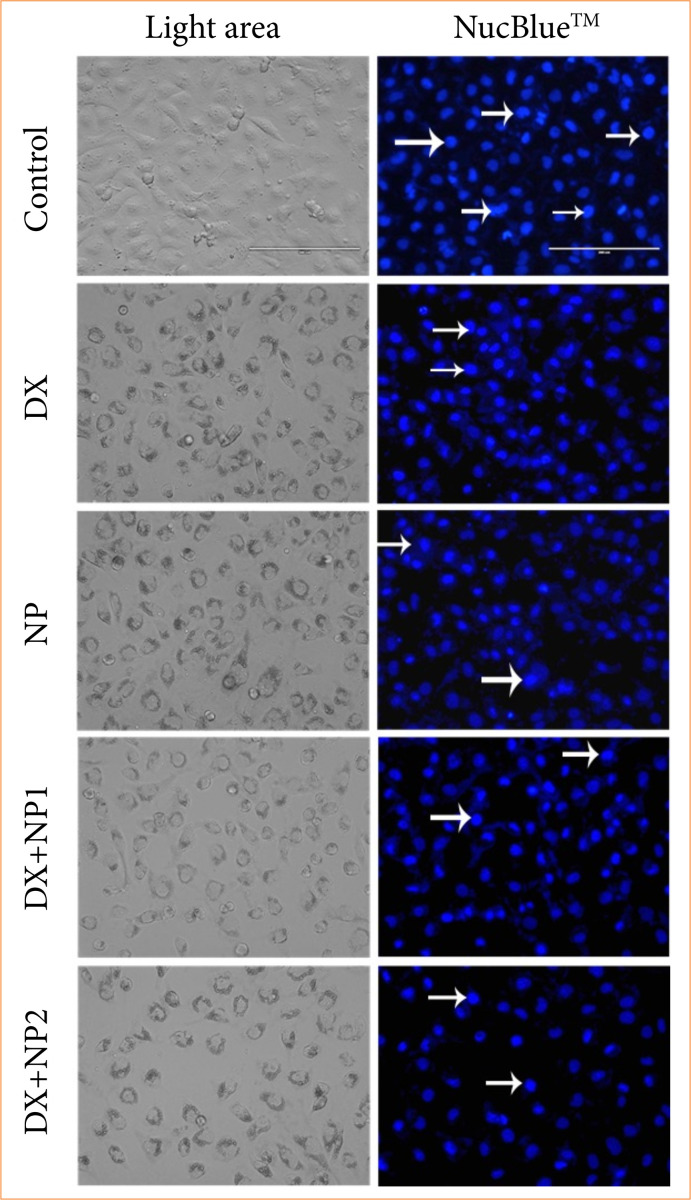
Vehicle control, SH-SY5Y neuroblastoma treated with doxorubicin (DX) IC50 0.769 μM, napabucasin (NP) IC50 2.1 μM, DX + NP1 (0.7 + 0.3 µM), and DX + NP2 (0.7 + 1 µM) for 48-hours cell morphology, nuclear structure and apoptotic body formation in cell populations. Arrow: living cell.

Source: Elaborated by the authors.

In the study, nuclear morphology changes and apoptotic structures occurring after apoptosis caused by DX and NP agents in the SH-SY5Y cell line are given in Fig. 4 with NucBlue Live ReadyProbes Reagent (ThermoScientific, United States of America) specific staining photographs. DNA differentiation in SH-SY5Y cells treated with NP and DX was determined by NucBlue Live ReadyProbes Reagent (ThermoScientific, United States of America) staining. Although there was an increase in the number of apoptotic cells in the groups administered only DX and only NP, the highest apoptotic activity was obtained in the NP + DX combination.

### qRT-PCR gene expression

**Figure 5:**
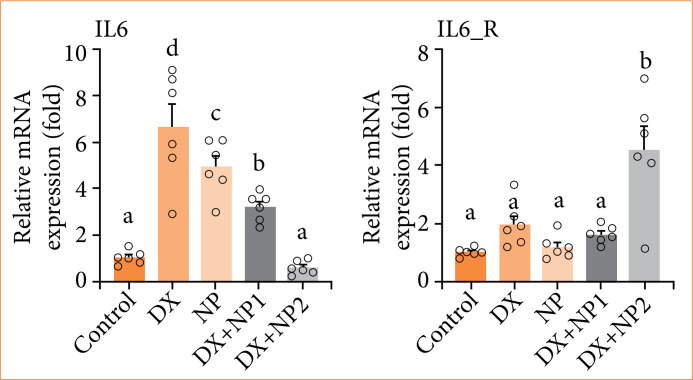
Relative fold increase values of interleukin (IL)-6/IL-6 R gene expressions 48 hours after single and combined application of doxorubicin (DX) and napabucasin (NP) in SH-SY5Y neuroblastoma cell series (data normalized with β-actin and GAPDH mRNA level by multiple control method, n = 6 data mean ± standard deviation). Means indicated with different letters are statistically different, one-way analysis of variance, Tukey’s HSD test, *p* ≤ 0.05. DX = 0.769 µM, NP = 2.1 µM, DX + NP1 = 0.7 + 0.3 µM; and DX + NP2 = 0.7 + 1 µM.

Source: Elaborated by the authors.

**Figure 6:**
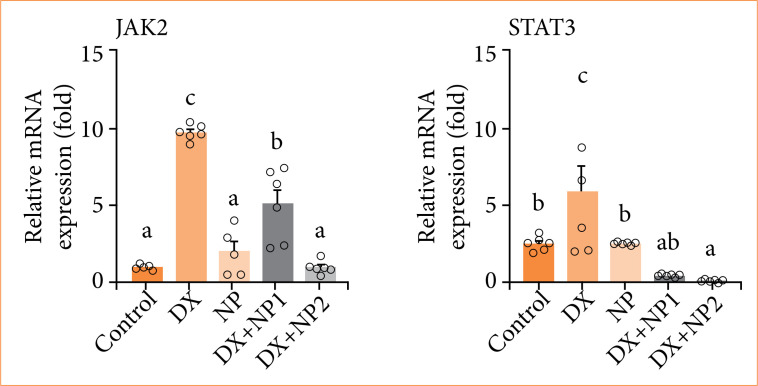
Relative fold increase values of Jak2/signal transducer and activator of transcription 3 (Stat3) gene expressions 48 hours after single and combined application of doxorubicin (DX) and napabucasin (NP) in SH-SY5Y neuroblastoma cell series (data normalized with β-actin and GAPDH mRNA level by multiple control method, n = 6 data mean ± standard deviation). Means indicated with different letters are statistically different, one-way analysis of variance, Tukey’s HSD test, *p* ≤ 0.05. DX = 0.769 µM, NP = 2.1 µM, DX + NP1 = 0.7 + 0.3 µM, and DX + NP2 0.7 + 1 µM.

Source: Elaborated by the authors.

In the scope of the study, vehicle control, DX and NP doses were applied individually and in combination to SH-SY5Y neuroblastoma cells, and IL-6, IL-6-R, Jak2, and Stat3 gene expressions were determined at the end of 48 hours. The effects of NP and DX on IL-6, Jak2, and Stat3 gene expressions are shown in Figs. 5 and 6. It was determined that, with NP application, IL-6 expression increased significantly and statistically compared to the control group (*p* < 0.001). Jak2 expression was not significant compared to the control group, and Stat3 expression increased, but not as significant as the control group.

In the DX applied group, IL-6, Jak2, and Stat3 expression was at the highest level of significance compared to the control group (*p* < 0.001), while the increase in IL-6 receptor was not significant. While IL-6 and Jak2 expression increased significantly in the DX + NP1 group (*p* < 0.01), Stat3 expression decreased significantly (*p* < 0.001). In the DX + NP2 group, although IL-6 and Jak2 decreased, there was no significance. The highest decrease in Stat3 expression was detected compared to the control group (*p* < 0.001) (Figs. 5 and 6).

### Western blot findings

**Figure 7:**
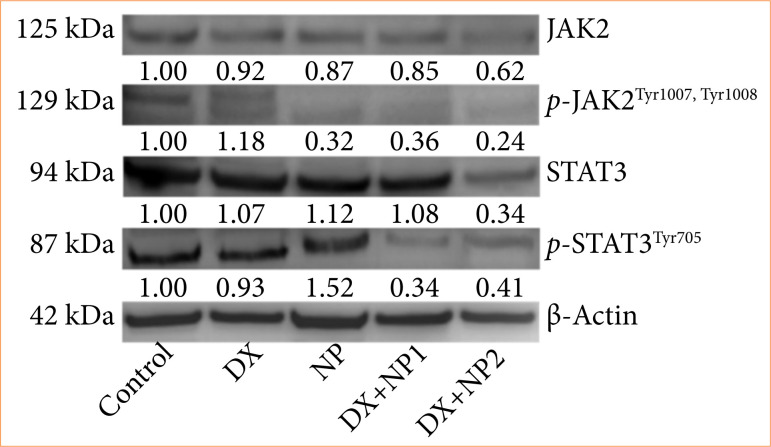
Vehicle control, doxorubucin (DX), and napabucasin (NP) were applied singly and in combination to SH-SY5Y neuroblastoma cells, JAK2, phospho-JAK2 (p Tyr1007, p Tyr1008), signal transducer and activator of transcription 3 (Stat3), phospho-STAT3 (p Tyr705) protein levels at the end of 48 hours. All data are in vehicle control. SH-SY5Y cell line is given as relative fold change according to target protein/actin = 1.

Source: Elaborated by the authors.

Napabucasin treatment (2,10 μM, 48 h) successfully impaired activation of the Jak2/Stat3 pathway. This observation was confirmed by a Western blot assay and qRT-PCR, demonstrating that treatment with NP reduced the protein levels of total and phospho-jak2/stat3 (Fig. 7). These data indicated that NP effectively restrained the hyperactivity of the Jak2/Stat3 pathway in SH-SY5Y cells.

## Discussion

In this study, we demonstrated that NP and DX effectively suppress the proliferation of neuroblastoma cells by targeting the Jak2/Stat3 signaling pathway. Our findings revealed that both agents individually and synergistically inhibit the activation of Jak2 and subsequent phosphorylation of Stat3, a critical transcription factor involved in promoting cell survival and proliferation in neuroblastoma. NP, known for its role in inhibiting cancer stem cell pathways, and DX, a widely used chemotherapeutic agent, were observed to decrease Stat3 activity, leading to reduced expression of downstream oncogenic targets. This suppression of the Jak2/Stat3 pathway resulted in significant growth inhibition and increased apoptosis in neuroblastoma cells.

Our results suggested that the combined use of NP and DX could be a promising therapeutic strategy for targeting the Jak2/Stat3 pathway in neuroblastoma, offering a potential avenue for enhancing effective treatment and overcoming resistance to conventional therapies. We showed that NP prevents the invasion and proliferation of neuroblastoma SH-SY5Y cells and that this effect is achieved by suppressing Jak2/Stat3 signaling pathway. With these results, we determined that NP, a natural phenolic compound, has protective roles in highly metastatic cancer types such as neuroblastoma.

Neuroblastoma is a complex disease affecting the sympathetic nervous system^13^. Most of the mortalities associated with neuroblastoma occur due to metastasis to lymph nodes and bones^14^^,^ 15. Therefore, preventing cancer cell invasion and migration is an important step to prevent metastasis^16^. Many studies have shown that the presence of neuroblastoma cells with unlimited regeneration and the capacity to regulate tumorigenic progenies are the main causes of disease relapse^17^. Therefore, efforts have been made to develop promising and effective strategies to target and eliminate these cells.

STAT3, an oncogenic transcription factor, is a promising target in cancer therapy. As it is the convergence point of many oncogenic signaling pathways, it is essential in controlling the immune response to antitumor agents. It is commonly hyperactivated in both cancerous and non-cancerous cells of the tumor structure. Many STAT3 inhibitor anticancer drugs such as rapamycin, angolin and hikonin have been proven^18^^–^ 20. Stat3 is an important transcription factor that can control many physiological and pathological processes, including cell proliferation, cell survival, and immune responses. Considering the role of overactivity of Stat3 signaling in neuroblastoma, the effectiveness of the therapeutic potential of targeting this pathway remains to be revealed. Many inhibitory agents have been developed against this signaling pathway, and the developed chemicals have been reported to have various successes, such as targeting Stat3-binding peptides and siRNA reagents^21^^,^ 22.

Li et al.^23^ described the newly discovered NP (BBI608) as a Stat3 inhibitor. They reported that NP can highly effectively suppress many cancer metastases and recurrences through inhibiting Stat3 gene transcription. Later, studies reported that NP treatment disrupted the spherical formation of cancer stem cells, which is the cause of metastasis in liver cancer, and by reducing the expression of stem cell genes such as SOX2, BMI-1, Nanog, and c-Myc.

Promising preclinical data of combinations of NP both as conventional chemotherapy and as monotherapy have attracted attention^24^. Moreover, the phase-III study of NP in resistant colorectal cancer highlighted Stat3 as a key target in patients with high pStat3 expression^25^. However, the effect of NP in neuroblastoma remains unclear. We confirmed that Stat3 is a factor that promotes malignant progression and poor prognosis of neuroblastoma. We observed that treatment of neuroblastoma cell line (SH-SY5Y) with NP and DX significantly blocked cell proliferation and migration. Additionally, we determined that NP has a high synergistic effect with DX and found that 48-hour NP + DX application inhibited cell proliferation through the Jak2/Stat3 signaling pathway. Protein and mRNA expression of Stat3 decreased following NP treatment.

NP treatment also significantly reduced the expression and phosphorylation of Stat3, a key transcription factor of the Jak2 signaling pathway. Additionally, NP offered advantages such as reducing the protein level of Stat3 and not only inhibiting the phosphorylation of STAT3. Zuo et al.^22^ reports that down-regulation of Stat3 protein level is due to NP-induced protein synthesis inhibition. In this study, we revealed that NP and DX treatment reduced Jak2/Stat3 expression at the transcriptional level. Our results support the potential use of NP as an effective anti-cancer therapeutic agent.

## Conclusion

The results showed that NP and DX suppressed Jak2/Stat3 expression and inhibited the proliferation and invasion of dSH-SY5Y cells. We also found that NP significantly reduced the expression and phosphorylation of Jak2/Stat3, a novel pharmacological mechanism. Therefore, the combination of NP and DX may be a promising agent to suppress neuroblastoma progression.

## Data Availability

The data will be available upon request.
